# Exploring Ultrasound, Microwave and Ultrasound–Microwave Assisted Extraction Technologies to Increase the Extraction of Bioactive Compounds and Antioxidants from Brown Macroalgae

**DOI:** 10.3390/md18030172

**Published:** 2020-03-20

**Authors:** Marco Garcia-Vaquero, Viruja Ummat, Brijesh Tiwari, Gaurav Rajauria

**Affiliations:** 1 School of Agriculture and Food Science, University College Dublin, Dublin 4 Belfield, Ireland; marco.garciavaquero@ucd.ie; 2 TEAGASC Food Research Centre, Dublin 15 Ashtown, Ireland; viruja.ummat@ucdconnect.ie (V.U.); brijesh.tiwari@teagasc.ie (B.T.); 3 School of Biosystems and Food Engineering, University College Dublin, Dublin 4 Belfield, Ireland

**Keywords:** fucoidan, carbohydrate, polyphenol, antioxidant, innovative technology, functional food, biorefinery

## Abstract

This study aims to determine the influence of (1) ultrasound-assisted extraction (UAE), (2) microwave-assisted extraction (MAE) and (3) a combination of ultrasound–microwave-assisted extraction (UMAE) on the yields of fucose-sulphated polysaccharides (FSPs), total soluble carbohydrates and antioxidants extracted from *A. nodosum*. Scanning electron microscopy (SEM) was used to evaluate the influence of the extraction technologies on the surface of macroalgae while principal component analysis was used to assess the influence of the extraction forces on the yields of compounds. UMAE generated higher yields of compounds compared to UAE and MAE methods separately. The maximum yields of compounds achieved using UMAE were: FSPs (3533.75 ± 55.81 mg fucose/100 g dried macroalgae (dm)), total soluble carbohydrates (10408.72 ± 229.11 mg glucose equivalents/100 g dm) and phenolic compounds (2605.89 ± 192.97 mg gallic acid equivalents/100 g dm). The antioxidant properties of the extracts showed no clear trend or extreme improvements by using UAE, MAE or UMAE. The macroalgal cells were strongly altered by the application of MAE and UMAE, as revealed by the SEM images. Further research will be needed to understand the combined effect of sono-generated and microwave-induced modifications on macroalgae that will allow us to tailor the forces of extraction to target specific molecules.

## 1. Introduction

Seaweed or macroalgae, with over 10,000 species, have been highlighted as promising sources of novel molecules and bioactive compounds in recent years [1,2]. Macroalgae are able to adapt to extreme environmental conditions by producing molecules such as polysaccharides, phenolic compounds, vitamins and minerals [1]. There is an increased interest in extracting these compounds for their use as pharmaceuticals, functional foods and nutraceuticals. Nutraceuticals are compounds with health benefits beyond those of basic nutrition when included in food products, offering possibilities in the prevention of pathological conditions in subjects not yet eligible for conventional pharmacological treatments [2,3]. Polysaccharides from macroalgae have been explored as nutraceuticals due to their wide range of biological properties including antioxidants [1,2,4]. There is evidence correlating the overproduction of reactive oxygen species (ROS) with ageing and multiple chronic diseases such as cancer, cardiovascular diseases and neurodegenerative disorders [5]. The recent literature has focused on the isolation of dietary antioxidants from multiple sources, including macroalgae with a strong antioxidant capacity against several ROS [6,7,8].

Polysaccharides, such as carrageenan, agar and alginates, have been traditionally used as gelling, thickening and stabilising agents in food, cosmetic and pharmaceutical formulations [9]. Moreover, recent studies have focused on the biological activities of macroalgal polysaccharides, i.e. soluble carbohydrates, such as glucans used as a feed additive, have shown promising properties, including anti-inflammatory activities when included in the diet of grower and finishing pigs [10,11]. Other polysaccharides, such as fucose-sulphated polysaccharides (FSPs), have shown beneficial effects when used as antioxidants, anti-inflammatory agents, anticoagulants and antivirals amongst other biological properties [4].

Phenolic compounds from macroalgae are part of a diverse and complex group that encompasses simple molecules such as phenolic acid, as well as more complex polymeric structures such as phlorotannins, with up to eight interconnected phloroglucinol units [12,13]. Multiple in vitro studies have demonstrated the efficiency of macroalgal phenolic compounds as antioxidant, antimicrobial and anticancer agents [7,14,15,16,17,18].

The biological activities of the macroalgal compounds, such as polysaccharides and phenolic compounds, are related to the chemical structure of the molecules, which is related to parameters affecting the macroalgal biomass (i.e., species, season and location of harvesting) [19] and the extraction procedures and purification conditions used [4,20]. Recent trends in algal biotechnology recommend exploring the use of innovative technologies individually, sequentially or simultaneously to increase the yields of bioactive compounds and to improve the efficiency of the extraction procedures, while reducing the use of chemicals, consumption of energy and generation of waste [21,22]. Innovative technologies, such as ultrasound-assisted extraction (UAE) and microwave-assisted extraction (MAE), have been recently explored to extract multiple bioactive compounds from macroalgae, as reviewed by Garcia-Vaquero et al. [4] and Tiwari [23]. Thereby, the use of UAE processes alone [24,25,26] or sequentially with other extraction technologies [9] has shown promising advantages when extracting polysaccharides such as FSPs, glucans and other antioxidant compounds from macroalgae. Moreover, the extraction of carbohydrates and other antioxidant compounds have also been explored using MAE from terrestrial plants [27,28] and macroalgae [14,29]. Understanding the impact of multiple process parameters on the efficiency of the extraction and on the yield of carbohydrates and other high-value compounds obtained from macroalgae is one of the key critical points that will influence the design and utilisation of macroalgal biomass following a biorefinery concept [9]. However, despite being some of the most promising novel technologies, to our knowledge, there are currently no studies researching the simultaneous application of ultrasounds and microwaves to generate high-value compounds from macroalgae.

Therefore, this study aims to determine the influence of ultrasounds, microwave and both extraction forces combined by using (1) ultrasound-assisted extraction (UAE), (2) microwave-assisted extraction (MAE) and (3) ultrasound–microwave-assisted extraction (UMAE) for short extraction times (2 and 5 min) on the yields of bioactives (FSPs, total soluble carbohydrates and phenolic compounds) and antioxidant activities (DPPH and FRAP) from brown macroalgae *A. nodosum*.

## 2. Results and Discussion

### 2.1. Proximate Composition of Initial Macroalgae

The proximate composition of the dried and milled macroalgae, previous to the application of any extraction procedure is: dry matter (91.89 ± 0.01%), ash (19.97 ± 0.12%), FSPs (6785.02 ± 43.90 mg fucose/100 g dried macroalgae (dm)), total soluble carbohydrates (11960.74 ± 140.56 mg glucose equivalents/100 g dm) and total phenolic compounds (1354.48 ± 36.33 mg gallic acid equivalents/100 g dm). The antioxidant properties of the full biomass were DPPH (79.55±0.68% radical scavenging activity) and FRAP (369.85 ± 10.17 µM trolox/mg freeze-dried extract (fde)).

### 2.2. Extraction Conditions

**Figure 1:**
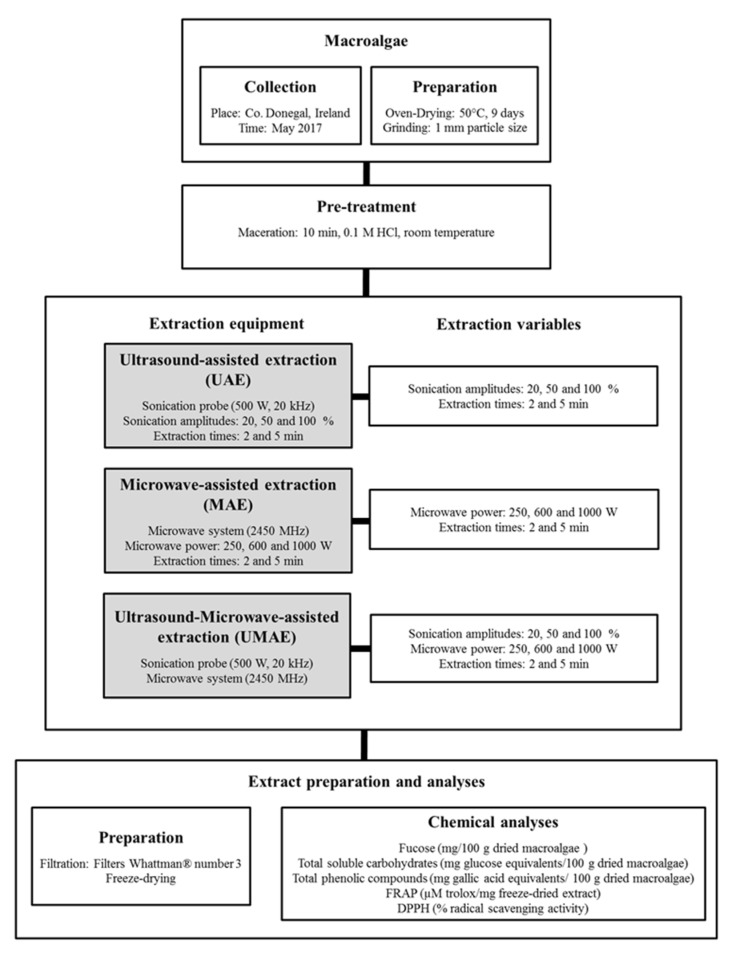
Scheme summarizing the preparation, pre-treatments and extraction conditions applied to *A. nodosum* to generate macroalgal extracts together with the chemical analyses performed.

The procedures followed to generate extracts by UAE, MAE and UMAE together with the analytical procedures employed to analyse the yields of FSPs, total soluble carbohydrates, phenolic compounds and associated antioxidant activities (DPPH and FRAP) are represented in Figure 1, with further details explained in the Materials and Methods (Section 3.3).

#### 2.2.1. Ultrasound-Assisted Extraction (UAE)

**Figure 2:**
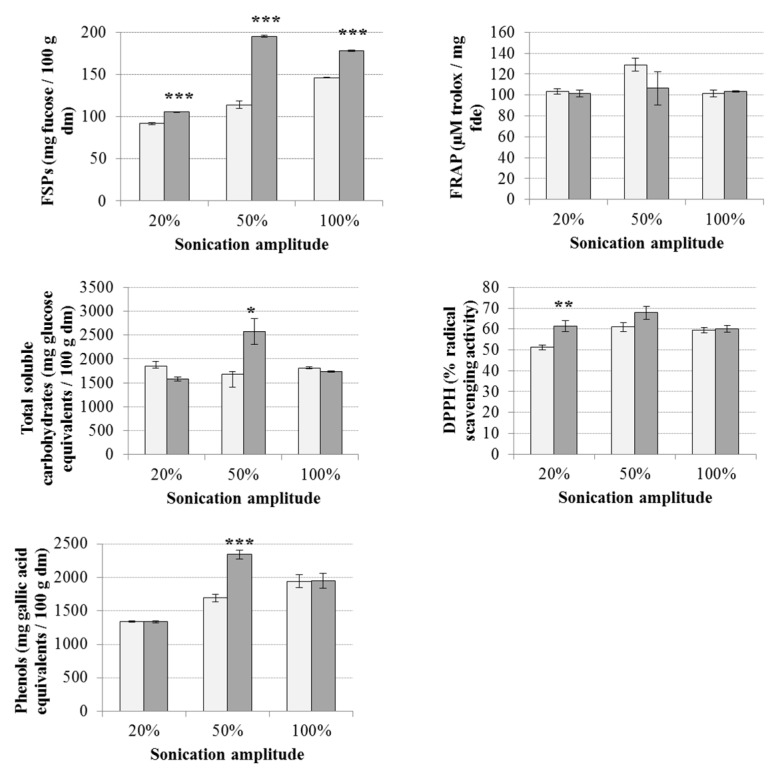
Yields of fucose-sulphated polysaccharides (FSPs), total soluble carbohydrates, phenolic compounds and antioxidant activities (FRAP and DPPH) of extracts from *A. nodosum* obtained by UAE at ultrasonic amplitudes (20%, 50% and 100%). Light and dark bars represent ultrasound-assisted extraction (UAE) treatments of 2 or 5 min, respectively. Results are expressed as average ± standard deviation of the mean (*n* = 6). The statistical differences between different treatment times for each UAE combination are expressed as follows: * *P* < 0.05, ** *P* < 0.01, *** *P* < 0.001.

The ultrasonic amplitude and time of UAE influenced the extraction yields of FSPs, total soluble carbohydrates, phenolic compounds and related antioxidant activities (Figure 2). There was a statistically significant (*P* < 0.001) increase in the yield of FSPs extracted from macroalgae after receiving UAE for 5 min compared to the yield obtained for 2 min treatment. Statistical differences were also appreciated for the extraction time when using 50% of ultrasonic amplitude in the case of total soluble carbohydrates and phenols and at 20% of amplitude for DPPH, while no statistical differences in treatment time were appreciated for FRAP.

The highest yields of FSPs (195.36 ± 1.36 mg fucose/100 g dm), total soluble carbohydrates (2572.97 ± 269.37 mg glucose equivalents/100 g dm), phenolic compounds (2340.54 ± 65.55 mg gallic acid equivalents/100 g dm), FRAP (128.82 ± 6.11 µM trolox/mg fde) and DPPH (67.81 ± 3.08% radical scavenging) were achieved using 50% of sonication amplitude for 5 min. The levels of FSPs and total carbohydrates extracted in this study were lower than in previous reports [24,26]; although these studies normally used higher temperatures and times of extraction compared to this study and other parameters, such as the technology and experimental conditions, may significantly influence the results. Kadam et al. [26] reported 87.06 mg fucose per g of extract from *A. nodosum* using UAE for 25 min at room temperature. Garcia-Vaquero et al. [24] obtained 2268.9 mg fucose/100 g dm using UAE at 80 °C, 30 min and 40% of ultrasonic amplitude, while the optimum extraction conditions to extract other carbohydrates such as glucans were obtained at UAE conditions of 52.5 °C, 10 min and 100% amplitude. The application of medium ultrasonic amplitudes was also described by Kadam et al. [25]. These authors achieved approximately 5.82% glucans (soluble carbohydrates) from *A. nodosum* by using UAE at 60% of ultrasonic amplitude for 15 min. In the case of phenolic compounds, the results obtained in this study were similar or even higher than previous reports using UAE. Agregán et al. [30] used UAE during 30 min combined with organic solvents (50% ethanol) to extract phenolic compounds and antioxidant extracts from several macroalgae and obtained a recovery of polyphenols of 4.66 g of phloroglucinol equivalents /100 g dm from *A. nodosum*. Moreover, the application of an optimised UAE protocol to achieve extracts with maximum antioxidant power from macroalgae (40 °C, 30 min and 40% of amplitude) achieved extracts from *A. nodosum* with similar antioxidant activities to those in this study with FRAP (160.4 ± 5.1 µM trolox/mg fde) and DPPH (87.0 ± 1.2%) [24]. However, the macroalgal species and season of collection of the biomass have an important role when comparing the extraction of bioactive compounds from macroalgae [4]. Thereby, Parys et al. [31] determined seasonal variations in the quantitative and qualitative contents of phenolic compounds from *A. nodosum*, being at its maximum in July; and Fletcher et al. [19] reported low levels of fucoidan in *A. nodosum* collected in March.

#### 2.2.2. Microwave-Assisted Extraction (MAE)

**Figure 3:**
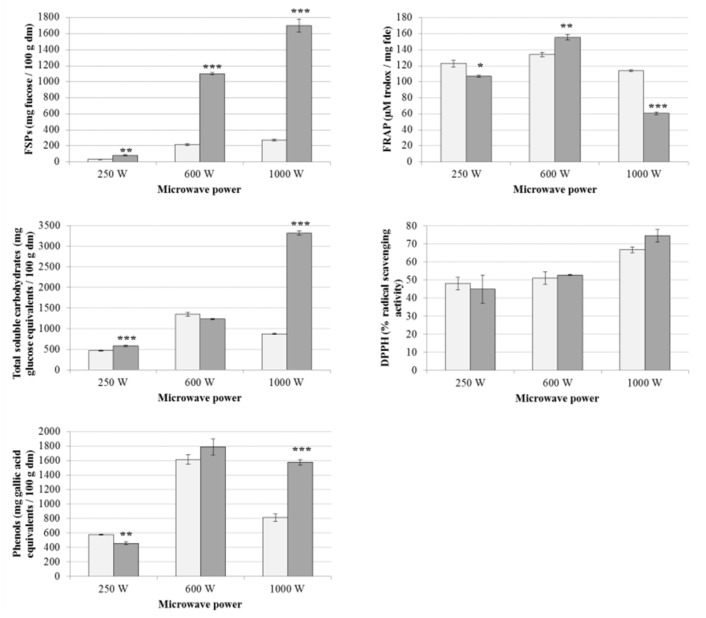
Yields of FSPs, total soluble carbohydrates, phenolic compounds and antioxidant activities (FRAP and DPPH) of extracts from *A. nodosum* obtained by microwave-assisted extraction (MAE) at microwave powers (250, 600 and 1000 W). Light and dark bars represent MAE treatments of 2 or 5 min, respectively. Results are expressed as average ± standard deviation of the mean (*n* = 6). The statistical differences between different treatment times for each MAE combination are expressed as follows: * *P* < 0.05, ** *P* < 0.01, *** *P* < 0.001.

When applying MAE, extraction times of 5 min at any microwave power studied (250, 600 and 1000 W) resulted in extracts containing statistically higher levels of FSPs compared to the same treatments applied for 2 min (see Figure 3). Variable influences of MAE time were also appreciated for the remaining compounds analysed, except in the case of DPPH antioxidant activities that did not show statistical differences depending on the extraction times.

The yields of FSPs obtained using MAE were approximately 10-fold higher than the maximum levels obtained using UAE. The maximum levels of FSPs (1699.80 ± 83.80 mg fucose/100 g dm) and total soluble carbohydrates (3317.39 ± 54.91 mg glucose equivalents/100 g dm) were obtained by using 1000 W of microwave power during 5 min, while the maximum yields of phenolic compounds (1790.93 ± 112.11 mg gallic acid equivalents/100 g dm) were achieved by applying 600 W of microwave power for 5 min. The antioxidant activities of the extracts were slightly improved with respect to UAE, although no clear MAE condition had a significant effect on improving the antioxidant properties of the extracts. The minimum yields of all the compounds studied in the extracts were obtained using 250 W, except in the case of FRAP antioxidant activities, which were lower at 1000 W.

Previous studies used MAE at 120 °C for 15 min and achieved yields of 16.08% of FSPs from *A. nodosum* [32]. MAE at 120 psi of pressure for 1 min, using water as a solvent for extraction (solvent-to-sample ratio 25:1 w/v) achieved extracts from *Fucus vesiculosus* with 18.22% of FSPs [29]. Ren et al. [33] obtained optimum yields of carbohydrates (2.84 ± 0.09%) from brown macroalgae *Sargassum thunbergii* using optimised MAE conditions of 547 W and 80 °C for 23 min. Other studies also used MAE (45 GHz, 1 kW) to extract polysaccharides from green macroalgae *Ulva* spp., obtaining maximum yields of these compounds at 140 °C for 10 min [34]. Furthermore, the maximum phenolic compounds (1790.93 ± 112.11 mg gallic acid equivalents/100 g ds) achieved in the current study by applying 600 W for 5 min, were higher than previous extraction protocols available in the literature using MAE. Yuan et al. [14] used MAE at 110 °C for 15 min and 50% ethanol to obtain extracts from *A. nodosum* containing 139.80 ± 10.82 mg gallic acid equivalents per 100 g dm.

#### 2.2.3. Ultrasound–Microwave Assisted Extraction (UMAE)

**Figure 4:**
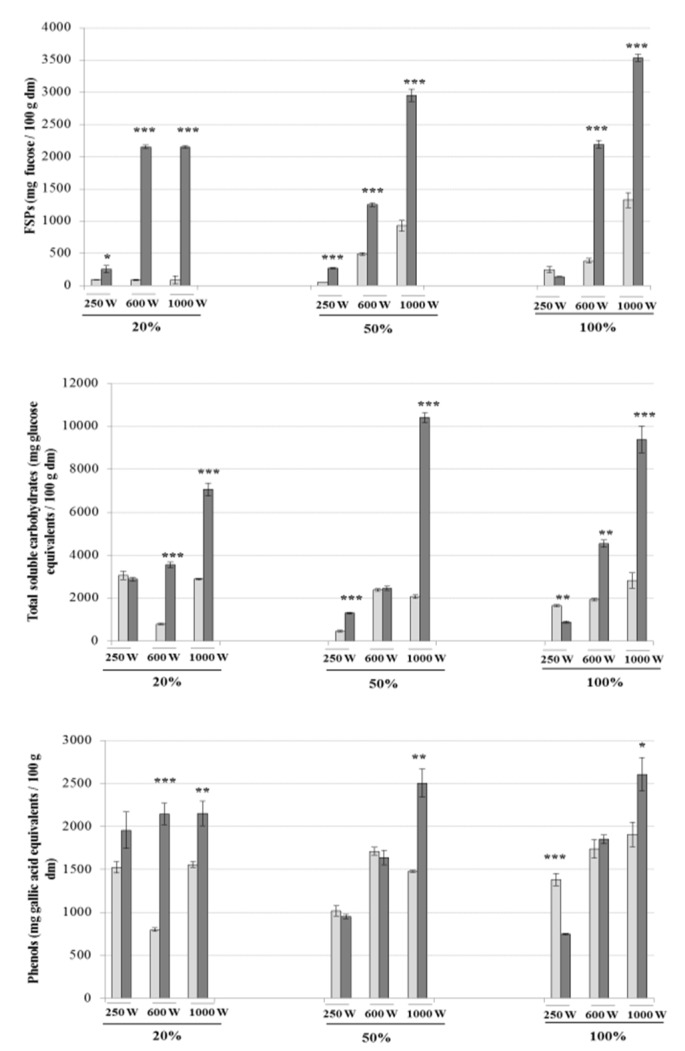
Yields of FSPs, total soluble carbohydrates and phenolic compounds of extracts from *A. nodosum* obtained by ultrasound–microwave-assisted extraction (UMAE). The recoveries were explored using multiple combinations of ultrasonic amplitude (20%, 50% and 100%) and microwave power (250, 600 and 1000 W). Light and dark bars represent the yields of each compound obtained when extracting macroalgae for 2 or 5 min, respectively. Results are expressed as average ± standard deviation of the mean (*n* = 6). The statistical differences between different treatment times for each UMAE combination are expressed as follows: * *P* < 0.05, ** *P* < 0.01, *** *P* < 0.001.

The simultaneous application of ultrasounds (20%, 50% and 100% sonication amplitude) and microwaves (250, 600 and 1000 W of microwave power) using UMAE and the effects of these treatment combinations on the yields of FSPs, total soluble carbohydrates and phenolic compounds are shown in Figure 4. The time of extraction significantly influenced the yields of FSPs, total soluble carbohydrates and phenols with maximum levels of FSPs (3533.75 ± 55.81 mg fucose/100 g dm) and phenolic compounds (2605.89 ± 192.97 mg gallic acid equivalents/100 g dm) obtained by using 100% of ultrasonic amplitude and 1000 W of microwave power during 5 min. The maximum yields of total soluble carbohydrates (10408.72 ± 229.11 mg glucose equivalents/100 g dm) were achieved by applying 100% of sonication amplitude and 600 W of microwave power for 5 min.

As seen by these results, the simultaneous application of microwave and ultrasounds had a significant effect on the yields of extraction of FSPs and total soluble carbohydrates. To our knowledge, this is the first study focusing on the simultaneous application of microwave and ultrasounds for extracting high-value compounds from macroalgae. Previous reports include the use of both forces to extract lipids from vegetables [35] and yeast [36]. Moreover, this combination of technologies was used to degrade phenolic compounds, as the sono-generated radicals in conjunction with the rapid thermal effect of microwaves have a significant effect when degrading polar chemicals [37].

**Figure 5:**
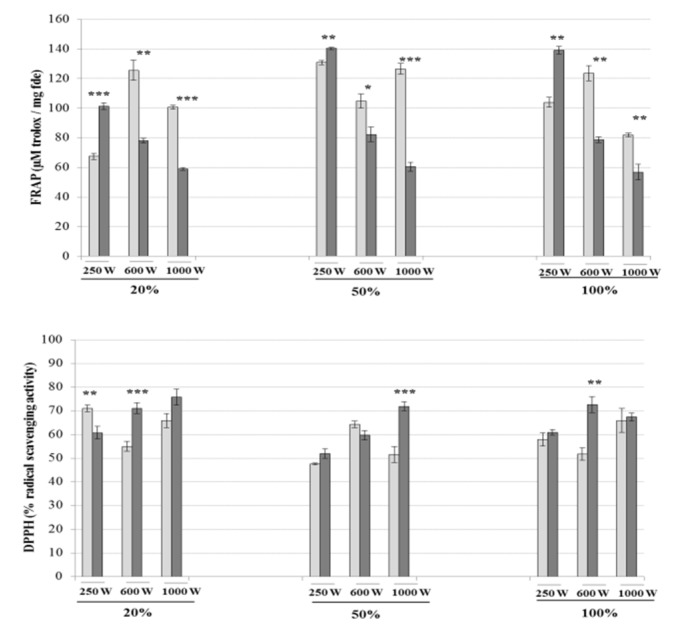
Antioxidant activities (FRAP and DPPH) of extracts from *A. nodosum* obtained by UMAE at multiple combinations of ultrasonic amplitude (20%, 50% and 100%) and microwave power (250, 600 and 1000 W). Light and dark bars represent the yields of each compound obtained when extracting macroalgae for 2 or 5 min, respectively. Results are expressed as average ± standard deviation of the mean (*n* = 6). The statistical differences between different treatment times for each UMAE combination are expressed as follows: * *P* < 0.05, ** *P* < 0.01, *** *P* < 0.001.

The influence of UMAE on the antioxidant activities of the extracts from *A. nodosum* is represented in Figure 5. The influence of the time of extraction, microwave power and sonication amplitude on the antioxidant activities of the macroalgal extracts were variable. The highest levels of FRAP (140.37 ± 0.79 µM trolox/mg fde) were obtained by applying simultaneously 250 W microwave power and 50% of ultrasound amplitude during 5 min, while the highest DPPH activities (75.86 ± 3.40%) were achieved in extracts obtained at 1000 W of microwave power, 20% sonication amplitude for 5 min.

**Figure 6:**
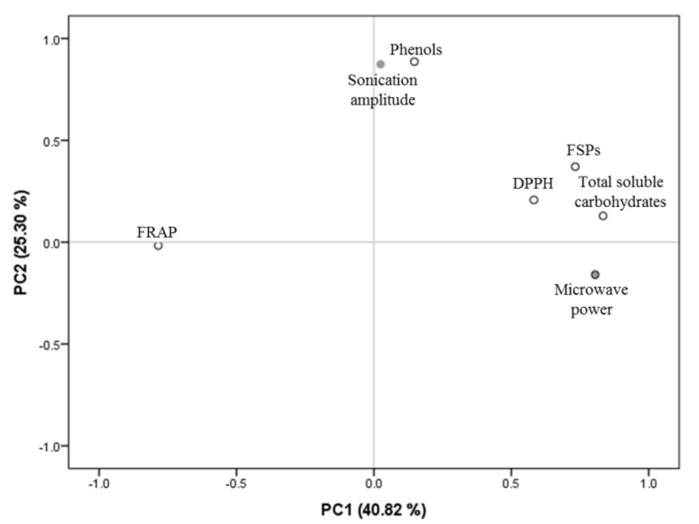
Principal component analysis scatter plot representing the scores for the extraction yields of FSPs, total soluble carbohydrates, phenols and antioxidant activities (FRAP and DPPH) of extracts from *A. nodosum* obtained using sonication and microwave technological treatments.

To understand the influence of sonication amplitude and microwave power on the extraction of FSPs, total soluble carbohydrates, phenolic compounds and antioxidant activities (FRAP and DPPH) from *A. nodosum*, all the extraction data were further analysed using principal component analysis (PCA). The two principal components, PC1 and PC2, obtained from the experimental data explained 40.82% and 25.30% of the total variance of the data set, respectively (see Figure 6).

PC1 explained 40.82% of the variance of the data and separates the yields of FSPs, total soluble sugars, phenolic compounds and DPPH on the right side of PC1, related also to the ultrasonic amplitude and microwave power. On the other hand, the FRAP antioxidant activities were situated on the opposite side of PC1 and could indicate a lower impact of the extraction parameters on the FRAP activity of the molecules obtained.

The second component explained further the variability of the data and separated two main groups in the data set. The yields of FSPs, total soluble carbohydrates and DPPH were situated in close proximity to the microwave power, while the yields of phenols were clustered together with the sonication amplitude further up in the positive side of PC2. These results could indicate the major influences of the different extraction forces to achieve high yields of different compounds. The different forces affecting the yields of phenolic compounds and carbohydrates may indicate the need to target phenolic compounds and carbohydrates separately in future biorefinery processes. Further studies will be needed in order to elucidate the impact of multiple extraction forces on the extraction of high-value compounds, allowing us to tailor the forces of extraction to target specific molecules, increasing the efficiency of extraction and the utilisation of macroalgae following a biorefinery process.

### 2.3. Scanning Electron Microscopy Analysis of Macroalgae

**Figure 7:**
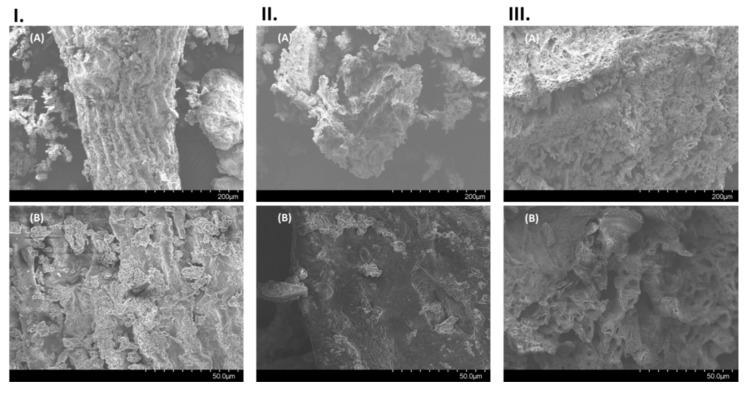
Scanning electron microscopy images of (**I**) dried and milled *A. nodosum* biomass before extraction, (**II**) macroalgal residue after MAE (250 W, 2 min) and (**III**) macroalgal biomass after the process of UMAE (1000 W, 100%, 5 min). Scale bars (A) 200 µm (magnification: 250×) and (B) 50 µm (magnification: 1000×).

Scanning electron microscopy (SEM) images were taken to understand the impact of the technological treatments on the macroalgal biomass. A similar methodology was used by Rodriguez-Jasso et al. [29] when evaluating the efficiency of MAE to generate extracts from the macroalga *Fucus vesiculosus*. Images of *A. nodosum* biomass previous to any extraction procedure together with the macroalgal residues from the extraction procedures achieving the lowest and highest yields of compounds MAE (250 W, 2 min) and UMAE (1000 W, 100% amplitude, 5 min), respectively, are presented in Figure 7.

The surface of the dried and milled *A. nodosum* biomass looks smooth and unaltered, as seen using a 250× magnification (Figure 7I.(A)). When increasing the magnification to 1000×, several impurities, together with salt residues, can be appreciated at the surface of the intact macroalgae (Figure 7I.(B)). The application of MAE (250 W, 2 min) achieved low yields of compounds (FSPs, total soluble carbohydrates and polyphenols) compared to the application of UAE or UMAE. The small changes in the surface of macroalgae after the application of MAE can be appreciated when compared with the full *A. nodosum*. However, major damage in the macroalgal biomass can be appreciated after applying UMAE using 100% ultrasonic amplitude and 1000 W of microwave power simultaneously for 5 min. The samples show high porosity (Figure 7III.(A),(B)), suggesting a combined effect of microwave radiation and ultrasound on the destruction of the cuticles of macroalgae and explaining the high yields of compounds extracted by the combination of extraction forces using UMAE.

## 3. Materials and Methods

### 3.1. Macroalgal Biomass Preparation and Composition Analyses

*A. nodosum* was harvested in Co. Donegal (Burtonport, Donegal, Ireland) in May 2017 by Quality Sea Veg Ltd. The macroalgae were cleaned of epitopes, oven-dried and milled using a Christy and Norris Hammer Mill (Chelmsford, UK). The samples were sieved to 1mm particle size using a sieve-shaker (VWR International LLC, Ireland) and vacuum-packed before the extraction. The dry matter of the macroalgae was determined by oven-drying the samples (105 °C, 16 h) and the ash content by igniting the samples in a muffle furnace (550 °C, 6 h) following the AOAC.942.05 [38]. The nitrogen content was determined using the LECO FP 528 instrument (Leco Instruments UKLTD., Cheshire, UK) and the protein contents were estimated using the conversion factor 4.17, as described for brown macroalgae by Biancarosa et al. [39]. The analyses of FSPs, total soluble sugars, total phenolic content and antioxidant capacity of the macroalgal biomass required preparation steps are described in detail in Section 3.4 and Section 3.5, respectively.

### 3.2. Chemicals

l-Fucose, ascorbic acid, d-glucose, phenol, gallic acid, Folin-Ciocalteu’s phenol reagent (2 M), citric acid, acetic acid, sodium hydroxide, sodium acetate, ferric chloride, sodium phosphate dibasic, methanol, triton™ X-100, sulfuric acid (95%–97%), l-cysteine, potassium hydroxide, hydrochloric acid, 1,1-diphenyl-2-picryl-hydrazyl (DPPH), 2,4,6-tripyridyl-*s*-triazine (TPTZ), 6-hydroxy-2,5,7,8-tetramethylchromane-2-carboxylic acid (trolox) were purchased from SIGMA (Sigma-Aldrich, Saint Louis, MO, USA). Ultrapure water was used in all the extraction procedures and chemical analyses.

### 3.3. Extraction Technologies and Procedures

**Figure 8:**
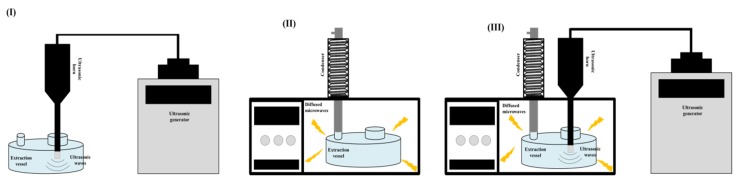
Schemes showing the technological designs (**I**) ultrasound-assisted extraction (UAE), (**II**) microwave-assisted extraction (MAE) and (**III**) ultrasound–microwave-assisted extraction (UMAE) used to generate macroalgal extracts.

The technological designs of UAE, MAE and UMAE used in this study to generate macroalgal extracts are represented schematically in Figure 8. UAE was performed using a UIP500hdT ultrasonic processor (500 W, 20 kHz, Hielscher Ultrasound technology, Teltow, Germany) at sonication amplitudes ranging from 20% to 100%. MAE was performed using a microwave oven (Panasonic NN-CF778S0, Bracknell, UK; 2450 MHz) at 250, 600 or 1000 W of nominal power. UMAE was performed using the aforementioned devices combined, allowing the simultaneous application of ultrasounds and microwaves to the sample. Multiple combinations of ultrasonic amplitude (20%, 50% and 100%) and microwave power (250, 600 and 1000 W) were explored using this technological design. The temperature of the initial mixtures was 22.77 ± 0.25 °C. As these extraction technologies do not allow us to control the temperature of the process, the temperatures after each extraction were monitored and recorded. The ranges of temperature after the extraction processes were as follows: UAE (23.3–30.5 °C), MAE (37.7–92 °C) and UMAE (36.2–98 °C).

The process of generating extracts from *A. nodosum* using multiple technologies is represented schematically in Figure 1. In general, macroalgal samples were macerated for 10 min using 0.1 M HCl with pH 1 (1:10, w/v) before starting the extraction procedures, as described in previous papers, focusing on achieving high yields of polysaccharides [10,25,26]. Multiple UAE, MAE and UMAE conditions were applied for short times (2 and 5 min) aiming to improve the efficiency of the extraction processes, similarly to previous reports using microwave technologies to obtain extracts from macroalgae [29]. The application of similar extraction times using UAE, MAE and UMAE allowed us to determine the influence of the time on the yields of extraction using each individual technology and to compare the yields obtained using the three innovative technological designs. Each extraction condition was performed in duplicate and the residual biomass was filtered through Whattman® number 3 (GE Healthcare, Buckinghamshire, UK). The supernatants of both extractions were combined, freeze-dried (FD80 model 119, Cuddon Engineering, Blenheim, New Zealand), vacuum sealed and stored at −20 °C for further analyses.

### 3.4. Composition and Antioxidant Analyses of Extracts

All the composition and antioxidant analyses of the extracts were performed in triplicate, with two readings of each replica (*n* = 6).

#### 3.4.1. Fucose Determination

FSPs were estimated based on the concentration of fucose in the macroalgal extract. The fucose content was determined as described in Garcia-Vaquero et al. [9]. Briefly, the samples, fucose standards (0.005 to 0.1 mg/mL) and H_2_O as negative control were mixed with 4.5 mL of 80% sulfuric acid solution and incubated for 10 min at 100 °C in a water bath. Cysteine hydrochloride (100 μL, 3%) was added to the hydrolysed samples and standards and further incubated for 60 min at room temperature. The reactions were read at 396 and 430 nm in polystyrene cuvettes in a spectrophotometer (Epoch, BioTek, Winooski, VT, USA). The values are expressed as mg fucose per 100 g of dried macroalgae (dm).

#### 3.4.2. Total Soluble Carbohydrates

The analyses of carbohydrates were performed following the phenol-sulfuric acid method with the modifications, as described by Masuko et al. [40]. Briefly, 30 µL of appropriately diluted samples (1mg/mL) or glucose standards (0–50 mg/mL) were added to each well in a 96 microplate (Greiner CELLSTAR^®^, Frickenhausen, Germany). A total of 150 µL of concentrated sulfuric acid was added to each well and mixed for 2 min followed by the addition of 30 µL of a 5% phenolic solution. The microplate was incubated in a water bath at 90 °C for 5 min, cooled down and the absorbance of the reaction read at 490 nm in a microplate reader (Epoch, BioTek, Winooski, VT, USA). The values of TSC are expressed as mg glucose equivalents per 100 g dm.

#### 3.4.3. Phenolic Compounds

The total phenolic content of the macroalgal extracts was determined using the Folin–Ciocalteau phenol reagent with the modifications described by Ganesan et al. [41]. An aliquot of 100 µL of diluted sample (1 mg/mL) or gallic acid standard (0–0.5 mg/mL) was mixed for 2 min with 2 mL of a 2% solution of sodium carbonate followed by the addition of 100 µL of 1 M Folin–Ciocalteau phenol reagent. The mixture was allowed to stand for 30 min at room temperature in dark conditions. The absorbance of the reaction was read at 720 nm in polystyrene cuvettes in a spectrophotometer. The total phenolic content of the extracts was expressed as mg gallic acid equivalents per 100 g dm.

### 3.5. Antioxidant Activity Analyses

#### 3.5.1. FRAP Assay

The ferric reducing antioxidant power (or FRAP assay) was performed following the method proposed by [42]. Briefly, 280 µL of a FRAP working solution containing acetate buffer (300 mM, pH 3.6), ferric chloride (20 mM in Milli Q water), 2,4,6-Tripyridyl-s-Triazine (TPTZ) (10 mM in 40 mM HCl) and Milli Q water, were added to 20 µL of macroalgal extracts (1 mg/mL) and trolox standards (15–420 µM). The mixtures were incubated at 37 °C for 30 min and the absorbance of the reaction was read at 593 nm. The FRAP antioxidant activity of each extract is expressed as µM trolox equivalents per mg of freeze-dried extract.

#### 3.5.2. DPPH Radical Scavenging Assay

The 2,2-diphenyl-1-picrylhydrazyl radical scavenging activity (or DPPH assay) was performed according to the method described by Nicklisch and Waite [43] modified by Garcia-Vaquero et al. [24]. Briefly, ascorbic acid used as positive control and macroalgal extracts were diluted to 1 mg/mL in 0.1 M citrate phosphate buffer with 0.3% of Triton X-100. An aliquot of 10 µL of a 2 mM methanolic DPPH solution was added to each well, mixed and incubated for 30 min at room temperature in dark conditions. The % of DPPH inhibitory activity was calculated by subtracting the absorbance readings of the wells at 515 nm before and after the reaction with the DPPH solution.

### 3.6. Scanning Electron Microscopy (SEM)

The shape and the surface characteristics of the full macroalgae and the samples achieving the highest and lowest yields of compounds after the process of extraction were collected and freeze-dried for SEM analysis. The macroalgal images were observed and recorded using a field emission scanning electron microscope Regulus 8230 (Hitachi Ltd., Tokyo, Japan).

### 3.7. Statistical Analyses

All the statistical analyses were performed in SPSS version 24.0. The influence of time on the recovery of FSPs, total soluble carbohydrates and total phenolic compounds and associated antioxidant activities was analysed using Student’s t-test. The variance in the data was analysed by principal component analysis (PCA) using Varimax rotation with Kaiser normalisation to obtain the expected weight for each component with eigenvalues higher than 1.

## 4. Conclusions

Overall, UAE, MAE and UMAE, applied in short timeframes (2 and 5 min), were able to extract FSPs, total soluble carbohydrates, total phenolic compounds, and antioxidant activities (FRAP and DPPH) from the brown macroalga *A. nodosum*. The yield of compounds and antioxidant properties improved by using MAE compared to UAE. The levels of FSPs obtained using MAE were approximately 10-fold higher than the maximum levels obtained using UAE. The application of MAE also achieved high yields of the other compounds studied: total soluble carbohydrates (3317.39 ± 54.91 mg glucose equivalents/100 g dm) and phenolic compounds (1790.93 ± 112.11 mg gallic acid equivalents/100 g dm). The simultaneous application of ultrasounds and microwaves by UMAE improved the yields of compounds extracted compared to the use of UAE and MAE alone. Maximum yields of compounds using several UMAE combinations were: FSPs (3533.75 ± 55.81 mg fucose/100 g dm), total soluble carbohydrates (10408.72 ± 229.11 mg glucose equivalents/100 g dm) and phenolic compounds (2605.89 ± 192.97 mg gallic acid equivalents/100 g dm). The antioxidant properties of the extracts showed no clear trend or extreme improvements by the use of UAE, MAE or UMAE which may be attributed to the co-extraction of other compounds contributing to the antioxidant properties of the extracts and not included in the scope of the present study. Moreover, a principal components analysis revealed the influence of microwave power on the yields of FSPs, total soluble carbohydrates and DPPH. To our knowledge, this is the first study focusing on the simultaneous application of microwaves and ultrasounds to extract high-value compounds from macroalgae. Future studies will be needed to confirm the antioxidant properties of the extracts in other biological models. Moreover, future technological improvements aiming to control extraction parameters such as temperature will be needed in order to apply high-intensity UAE and MAE combined in a more controlled manner, allowing us to explore and further understand the combined effect of sono-generated and microwave-induced modifications on macroalgal tissues that will allow us to tailor the forces of extraction to target specific molecules, increasing the efficiency of extraction and utilisation of macroalgae following a biorefinery process.
